# Causal effects of post-traumatic stress disorder on autoimmune thyroid disease: insights from mendelian randomization

**DOI:** 10.3389/fpsyt.2024.1417302

**Published:** 2024-09-20

**Authors:** Zhaorong Chen, Yunfeng Yu, Jiayu Yao, Zirui Guo, Yanhui Cui, Fang Li, Changqi Li

**Affiliations:** ^1^ Department of Anatomy and Neurobiology, School of Basic Medical Science, Central South University, Changsha, Hunan, China; ^2^ Graduate School, Hunan University of Chinese Medicine, Changsha, Hunan, China

**Keywords:** post-traumatic stress disorder, autoimmune thyroid disease, autoimmune thyroiditis, Graves’ disease, Mendelian randomization

## Abstract

**Objective:**

The relationship between post-traumatic stress disorder (PTSD) and autoimmune thyroid disease (AITD) needs further evaluation. This study employs Mendelian randomization (MR) to investigate the causal correlations of PTSD with autoimmune thyroiditis (AIT) and Graves’ disease (GD).

**Methods:**

Datasets for PTSD, AIT, and GD were obtained from FinnGen. The exposure-outcome causal relationship was assessed using inverse variance weighted, MR-Egger, and weighted median. Horizontal pleiotropy was evaluated through the MR-Egger intercept, heterogeneity was examined using Cochran’s Q test, and robustness was assessed via leave-one-out sensitivity analysis.

**Results:**

MR analysis indicated no significant causal relationship between PTSD and AIT (OR 0.920, 95% CI 0.832 to 1.017, *p* = 0.103), but a potential increase in the risk of GD associated with PTSD (OR 1.056, 95% CI 1.008 to 1.105, *p* = 0.021). MR-Egger intercept showed no horizontal pleiotropy (*p* > 0.05), and Cochran’s Q showed no heterogeneity (*p* > 0.05). Sensitivity analysis suggested the MR results were robust.

**Conclusions:**

Evidence of an MR association between genetic liability to PTSD and an increased risk of GD were provided, but no evidence of association between PTSD and AIT. The findings indicate that individuals with PTSD may have an increased likelihood of developing GD, underscoring the importance of further research to comprehend the intricate interplay between PTSD and thyroid disorders.

## 1 Introduction

Post-traumatic stress disorder (PTSD) is a chronic psychiatric condition that can develop in individuals exposed to traumatic events and has been described as “the complex somatic, cognitive, affective, and behavioral effects of psychological trauma” (1). PTSD in the general population was found to be 3.9%, exhibiting considerable variability among countries. Specifically, high-income countries demonstrated a higher prevalence rate of 5.0%, which was twice that observed in upper-middle income countries (2.3%) and lower-low middle income countries (2.1%) (2). Factors such as younger age, female gender, unemployment, lack of current marriage, lower educational attainment, and reduced household income were identified as associated with an increased likelihood of developing PTSD (2). Trauma is associated with a multitude of immune system changes and abnormalities that could increase the risk for autoimmune disorders. This association is reflected in the common comorbidity between PTSD and immune-related diseases (3). A study conducted on active-duty service members in the United States demonstrated that those with a history of PTSD had a 58 percent higher likelihood of developing autoimmune diseases, even after controlling for factors such as BMI, smoking status, and alcohol consumption (4). In another study involving 2,490 male Vietnam veterans, PTSD was found to be linked to a higher prevalence of self-reported autoimmune disorders (5). Furthermore, research from Sweden revealed an increased risk of subsequent development of autoimmune diseases associated with stress-related disorders among the general population (6). These findings suggest that PTSD may serve as a potential risk factor for autoimmune diseases and warrant further investigation into underlying biological mechanisms and potential strategies for risk mitigation.

Autoimmune thyroid diseases (AITD) are organ-specific autoimmune diseases affecting approximately 2-5% of the population which mainly includes Graves’ disease (GD) and autoimmune thyroiditis (AIT) (7). Abnormal interactions between thyrocytes, antigen-presenting cells, and T cells lead to an autoimmune reaction against thyroid antigens (8). The development of AITD is significantly influenced by environmental and hormonal factors that disrupt the intricate neuroendocrine-immune interactions in genetically susceptible individuals (9). PTSD has been associated with disruptions in both the endocrine and immune systems, potentially heightening the vulnerability to autoimmune disorders (4). These dysregulations manifest as perturbations in cortisol levels, increased inflammatory responses, alterations in gene expression within immune cells, and accelerated immune cell aging (10). These abnormalities are likely to contribute to inflammation and compromised immune functioning, which affects thyroid function (4, 9, 11, 12). PTSD has been linked to thyroid dysfunction in some studies (13, 14); But some studies did not find the same association between GD and stressful life events or PTSD (15–17). Therefore, the relationship between PTSD and AITD needs further evaluation.

Observational studies may be subject to residual confounding, selection bias, and reverse causality. However, Mendelian randomization (MR) study, an epidemiological method, could minimize potential bias due to confounding and reverse causation (18). Therefore, we used MR to investigate the relationship between PTSD and genetic liability to AITD in European ancestry individuals.

## 2 Materials and methods

### 2.1 Study design

**Figure 1:**
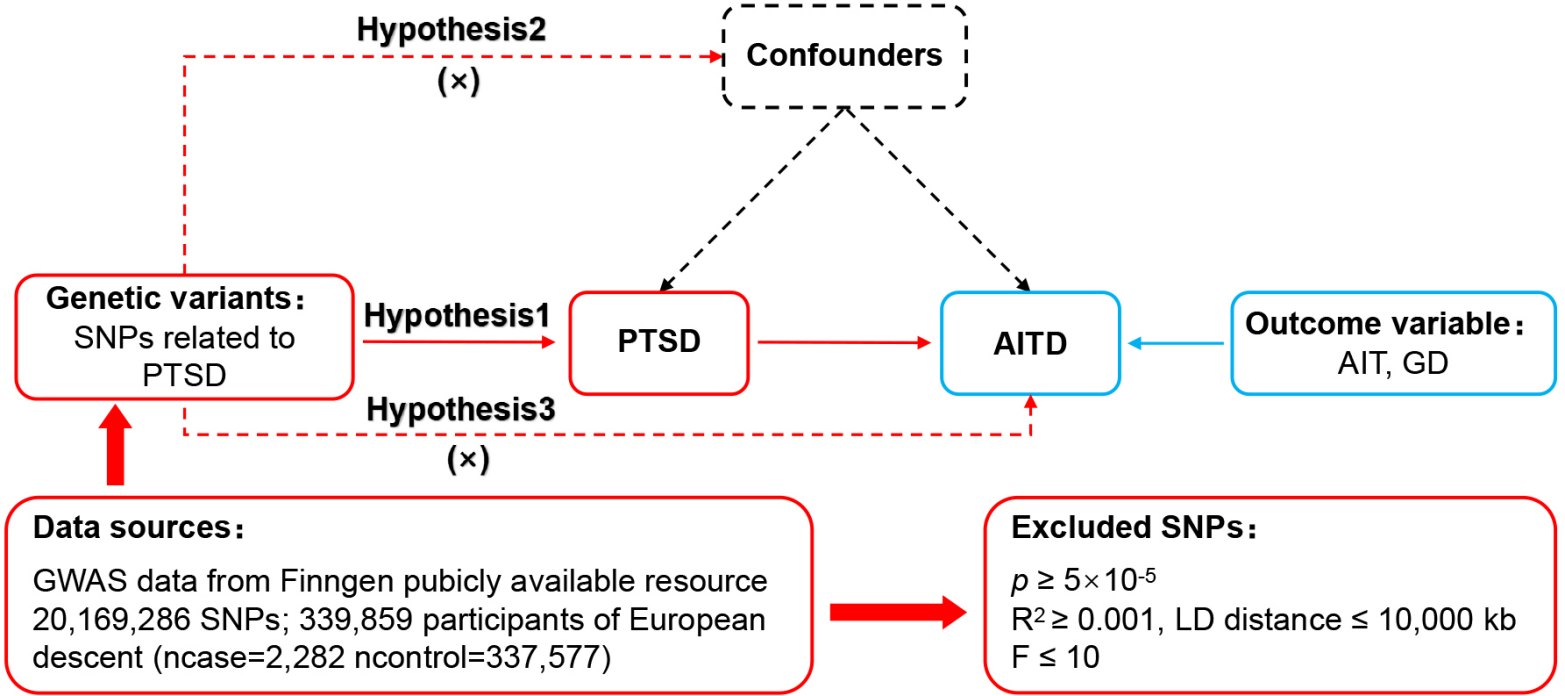
MR design for causal analysis of PTSD and AITD. PTSD, post-traumatic stress disorder; AITD, autoimmune thyroid disease; AIT, autoimmune thyroiditis; GD, Graves' disease.

MR relies on three core assumptions: (1) The relevance assumption (hypothesis 1): Single nucleotide polymorphisms (SNPs) were closely associated with the exposure factor (e.g. PTSD). (2) The independence assumption (hypothesis 2): SNPs were independent of confounding factors. (3) The exclusivity assumption (hypothesis 3): SNPs did not affect outcomes through non exposure pathways. The MR design process is shown in **Figure 1**.

### 2.2 Data sources

Datasets on PTSD, AIT, and GD used in this study were all sourced from the publicly available FinnGen (www.finngen.fi/fi), and each original dataset was approved by their institutional ethics committees.

### 2.3 Selection of SNPs

First, SNPs closely associated with PTSD were selected based on *p* < 5×10^-5^ to meet hypothesis 1. Second, independent SNPs were selected based on R^2^ < 0.001 and kb = 10,000 to avoid interference from linkage disequilibrium. Third, the F-value of each SNP was calculated and SNPs with F ≤ 10 were removed. The calculation formula for F-value is: $\text{F=}\left[{\text{R}}^{2}/\left(1-{\text{R}}^{2}\right)\right]*\left[\left(\text{N}-\text{K}-1\right)/\text{k}\right]\text{. }{\text{R}}^{2}=2*\left(1-\text{MAF}\right){\text{*MAF*β}}^{2}.\;{\text{R}}^{2}$ : the cumulative explained variance of the selected IVs on exposure; MAF: the effect of minor allele frequency; β: estimated effect of SNP; N: sample size. Fourth, PhenoScanner was consulted to exclude SNPs potentially related to AITD, satisfying hypotheses 2. Fifth, the direction of the exposure and outcome alleles was adjusted, and non-match SNPs were excluded based on the effect of allele frequency. Sixth, MR-Pleiotropy RESidual Sum and Outlier was used to remove outlier SNPs (*p* < 1.0), in order to ensure the correctness of causal inference.

### 2.4 Data analysis

This study adhered to the STROBE-MR (19). MR analysis was conducted using the “TwoSampleMR (0.5.7)” package in R 4.3.1, employing inverse variance weighted (IVW), MR-Egger, and weighted median as the methods for evaluating causal relationships. IVW served as the main analytical approach, while MR-Egger and weighted median were employed as supplementary analytical approach. To assess horizontal pleiotropy, the MR-Egger intercept was utilized, and a *p*-value of ≥ 0.05 indicated the absence of horizontal pleiotropy, thereby fulfilling hypothesis 3. Heterogeneity was evaluated using Cochran’s Q, and a *p*-value of ≥ 0.05 suggested no significant heterogeneity. To evaluate the robustness of the MR results and identify SNPs that have a significant impact on the outcomes, leave-one-out sensitivity analysis was performed.

## 3 Results

### 3.1 Data for exposure

The PTSD dataset included 339,859 individuals of European descent with dataset ID: FinnGen_R9_F5_PTSD. After excluding the effects of linkage disequilibrium and confounding factors, a total of 93 SNPs were included (see **Supplementary Table S1**). Subsequently, the non-match SNPs were removed based on the effect of allele frequency and outlier SNPs were excluded in the MR-Pleiotropy Residual Sum and Outlier. The final included SNPs are shown in **Supplementary Table S2**.

### 3.2 Data for outcome

The AIT and GD data used in this study were sourced from FinnGen and are shown in **Table 1**. The AIT dataset comprised 321,192 individuals of European descent with dataset ID: FinnGen_R9_E4_THYROIDITAUTOIM. The GD dataset included 377,277 individuals of European descent with dataset ID: FinnGen_R9_E4_GRAVES_STRICT.

**Table 1:** Detailed information on the datasets included in Mendelian randomization.

Year	Trait	GWAS ID	Population	Sample size	Web source
2023	PTSD	finngen_R9_F5_PTSD	European	339,859	www.finngen.fi/en
2023	AIT	finngen_R9_E4_THYROIDITAUTOIM	European	321,192	www.finngen.fi/en
2023	GD	finngen_R9_E4_GRAVES_STRICT	European	377,277	www.finngen.fi/en

PTSD, post-traumatic stress disorder; AIT, autoimmune thyroiditis; GD, Graves' disease.

### 3.3 MR analysis results

**Figure 2:**
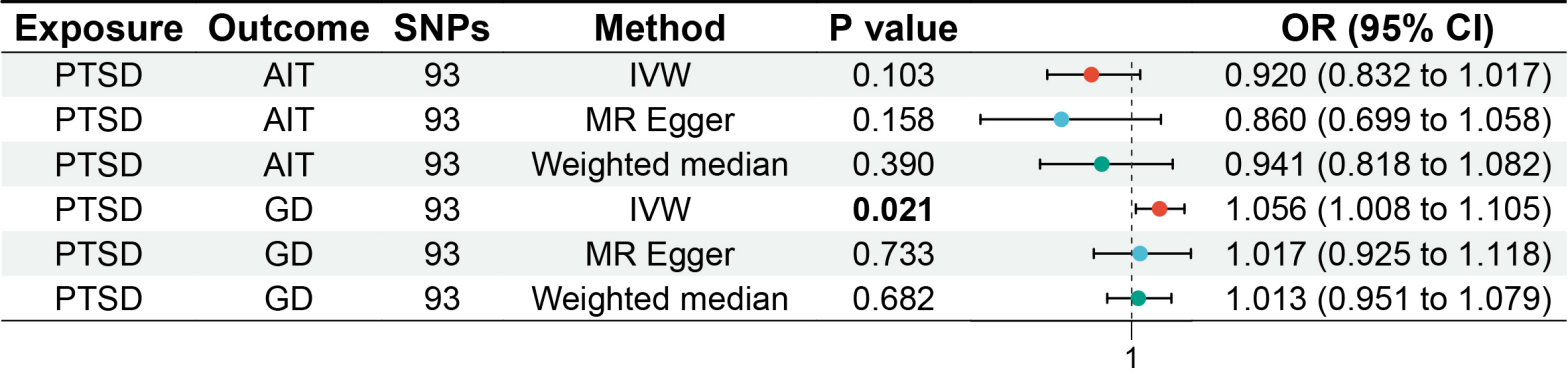
Forest plot of MR analysis on the causal relationship between PTSD and AITD. PTSD, post-traumatic stress disorder; AITD, autoimmune thyroid disease; AIT, autoimmune thyroiditis; GD, Graves' disease.

**Figure 3:**
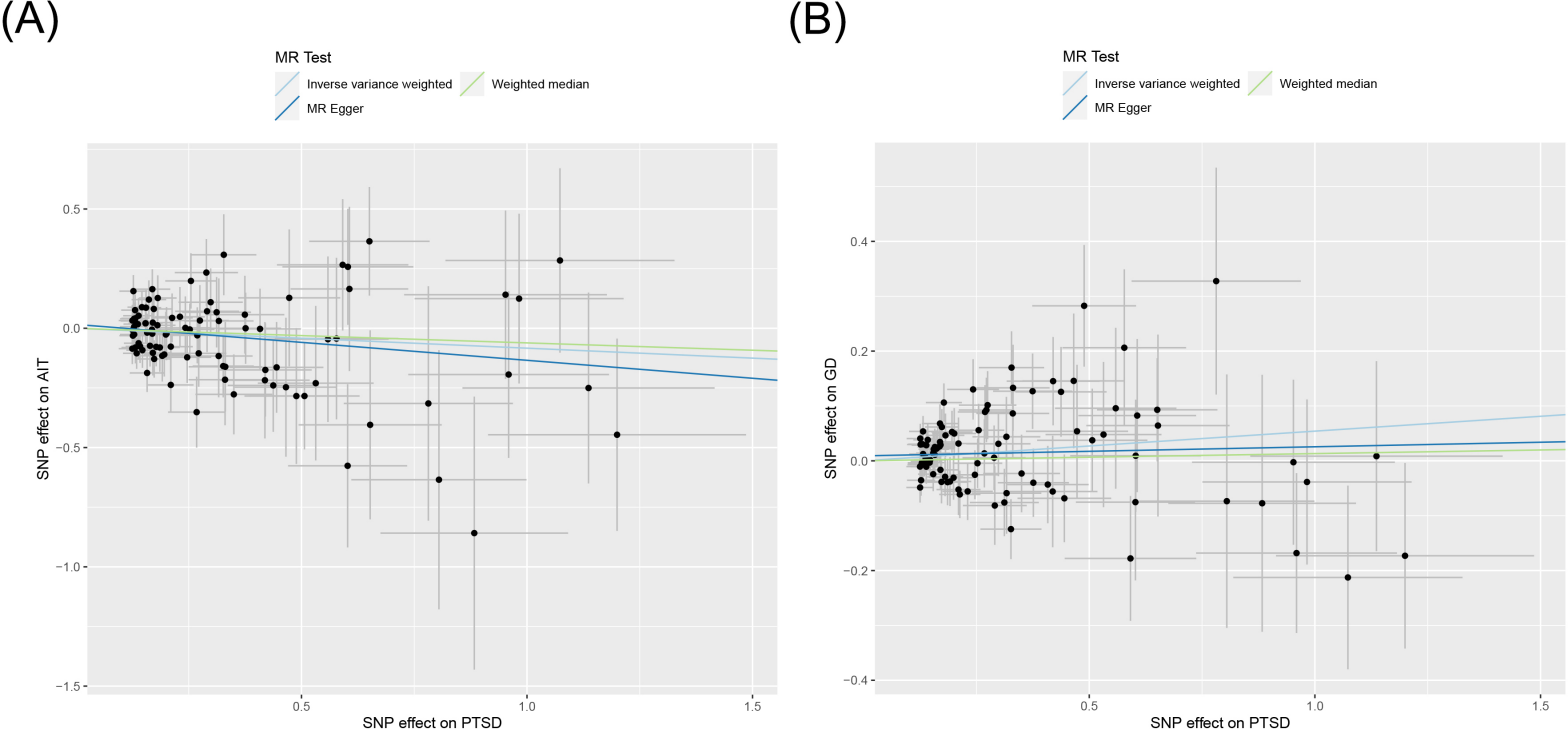
Scatter plot of MR analysis on the causal relationship between PTSD and AITD. (A) PTSD on AIT; (B) PTSD on GD. PTSD, post-traumatic stress disorder; AITD, autoimmune thyroid disease; AIT, autoimmune thyroiditis; GD, Graves' disease.

**Figure 4:**
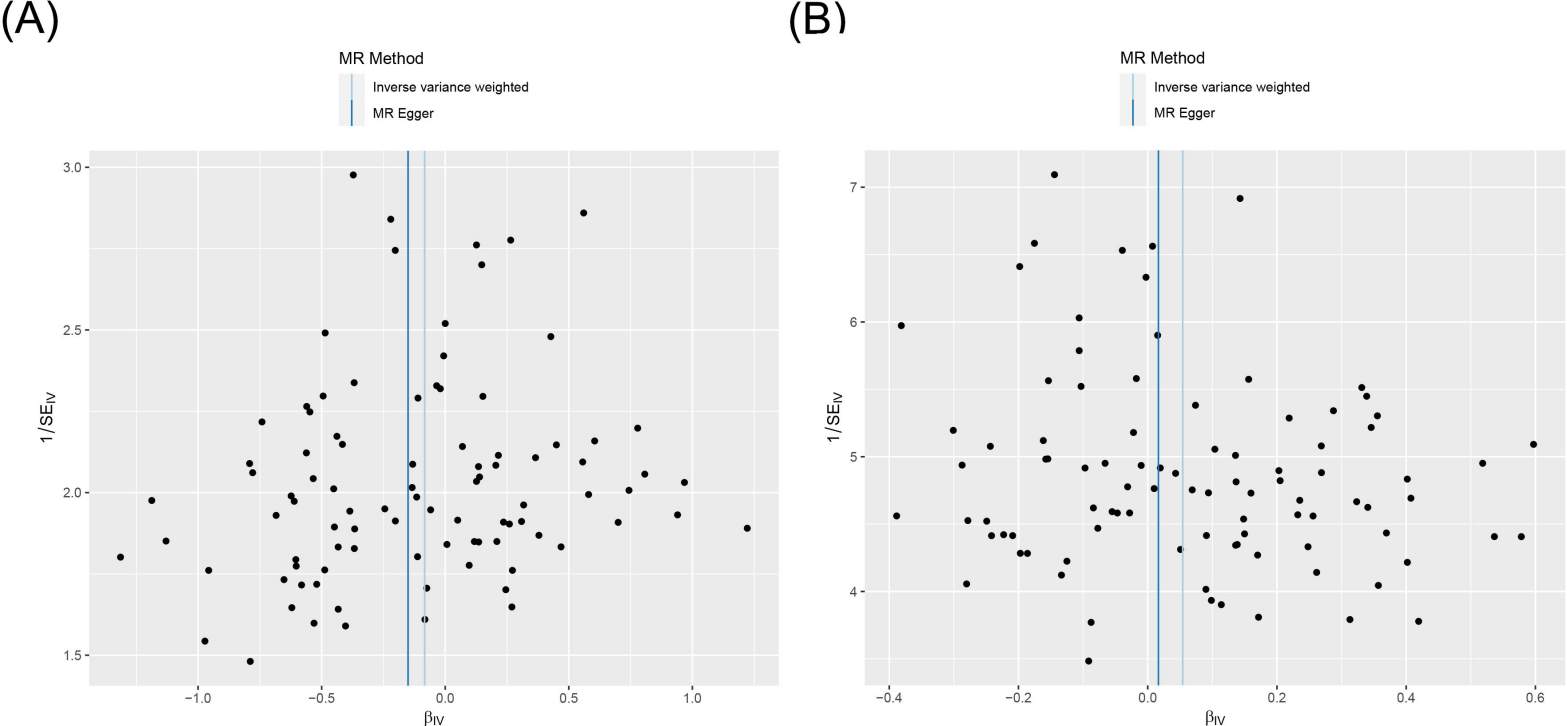
Funnel plot of heterogeneity analysis on the causal relationship between PTSD and AITD. (A) PTSD on AIT; (B) PTSD on GD. PTSD, post-traumatic stress disorder; AITD, autoimmune thyroid disease; AIT, autoimmune thyroiditis; GD, Graves' disease.

**Figure 5:**
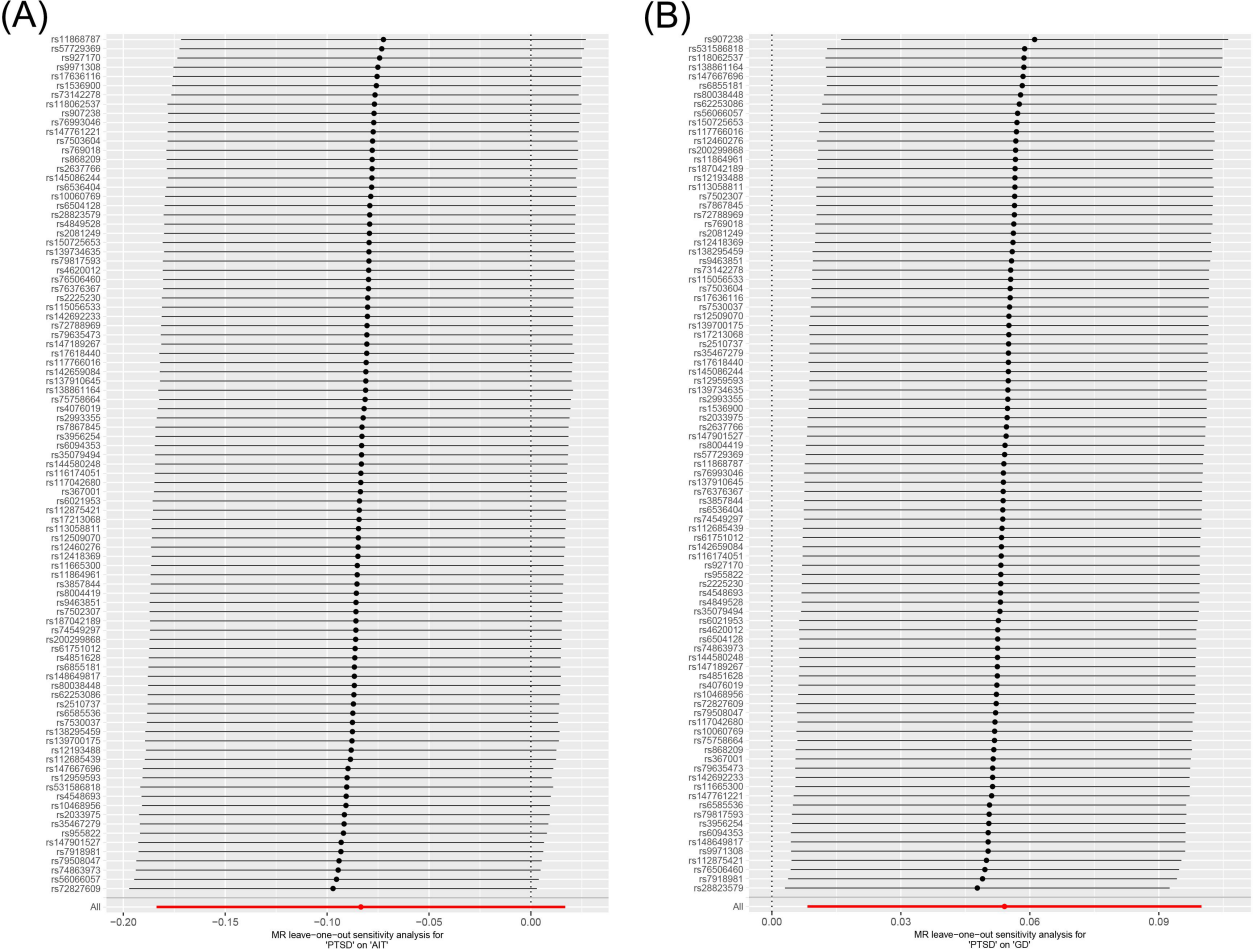
Leave-one-out sensitivity analysis on the causal relationship between PTSD and AITD. (A) PTSD on AIT; (B) PTSD on GD. PTSD, post-traumatic stress disorder; AITD, autoimmune thyroid disease; AIT, autoimmune thyroiditis; GD, Graves' disease.

MR analysis was conducted to examine the causal effects between PTSD and AITD. The forest plot of the MR analysis is presented in **Figure 2**, and the scatter plot can be found in **Figure 3**. The results of the horizontal pleiotropy are provided in **Supplementary Table S3**. The heterogeneity results are shown in **Figure 4**; **Supplementary Table S4**. Leave-one-out sensitivity analysis results can be found in **Figure 5**.

#### 3.3.1 Causal relationship between PTSD and AIT

All three analytical methods indicated no significant causal relationship between PTSD and AIT: IVW (OR 0.920, 95% CI 0.832 to 1.017, *p* = 0.103), MR Egger (OR 0.860, 95% CI 0.699 to 1.058, *p* = 0.158), weighted median (OR 0.941, 95% CI 0.818 to 1.082, *p* = 0.390), with no horizontal pleiotropy (*p* = 0.470). Cochran’s Q test demonstrated no heterogeneity (*p* = 0.402). Sensitivity analysis indicated robustness of the results.

#### 3.3.2 Causal relationship between PTSD and GD

IVW suggested a potential increase in the risk of GD associated with PTSD (OR 1.056, 95% CI 1.008 to 1.105, *p* = 0.021), whereas MR Egger (OR 1.017, 95% CI 0.925 to 1.118, *p* = 0.733) and weighted median (OR 1.013, 95% CI 0.951 to 1.079, *p* = 0.682) did not support this causal relationship. These results had no horizontal pleiotropy (*p* = 0.378). Cochran’s Q test indicated no heterogeneity (*p* = 0.066). Sensitivity analysis confirmed the robustness of the results.

## 4 Discussion

To our knowledge, this is the first MR study investigated the relationship between PTSD and genetic liability to AITD in European ancestry individuals. In evaluating the relationship between PTSD and the risk of AITD subtypes (GD and AIT), we found evidence of an association between PTSD and an increased genetic liability to GD, but no evidence of association between PTSD and AIT. These results were free of horizontal pleiotropy and heterogeneity, and sensitivity analysis suggested they were robust. The results conducted in this study adds significant evidence to the existing body of literature supporting PTSD as a potential risk factor for GD.

Prolonged exposure to stress is associated with dysfunction in the neuroimmune axis, leading to dysregulated immune cell responses and contributing to autoimmune diseases (20). Vitamin D regulates immune responses by activating T and B cells, macrophages, and dendritic cells, with its deficiency exacerbating inflammatory responses and playing a significant role in autoimmune conditions (21, 22). Additionally, chronic stress can alter gut microbiota, which in turn affects immunity (23). Vitamin D also enhances innate immune system function by regulating gut microbiota (24). IL-31 and IL-33 are emerging cytokines that play critical roles in regulating both adaptive and innate immune responses (25).

Although there is no direct research supporting the correlation between PTSD and GD, multiple epidemiologic studies have provided evidence supporting the correlation between stress and GD. Epidemiological observations have reported an increase in the incidence of GD during significant wars, such as the civil war in former Yugoslavia which witnessed a five-fold increase in GD compared to toxic nodular goiter (9). In addition, Sonino et (11), Winsa et al. (12), Kung et al. (26), Radosavljevic et al. (27), and Matos-Santos et al’s (28) studies revealed a higher frequency of negative and stressful life events in GD patients compared to controls. Further evidence from Vita et al’s prospective study found that all patients who relapsed GD had experienced at least one stressful event, and the total number of stressful events was significantly correlated with the frequency of relapses per patient (29). In a study of 293 GD patients treated with Iodine-131, those exposed to stress reached hypothyroid status earlier (30). A meta-analysis further indicated that stress is a significant factor in the onset of GD, with a high effect size (31). The investigators also reported a series of patients with stress-induced GD, even in the absence of antithyroid drugs (32). Previous studies and the results of this study all support that stress plays an important role in the pathogenesis of GD These findings highlight the importance of considering stress management strategies for individuals with GD and the need for further research to understand the relationship between stress and autoimmune thyroid diseases.

In contrast to GD, few studies focused on the role of stress on AIT, but the few epidemiological and clinical case-control studies, stress demonstrated no significant correlation with AIT development (9, 17, 33). Additionally, a separate study reinforced the absence of a triggering role for stress in AIT (34). A review summarizing the mediating role of environmental factors in the prevention of AITD also supports the notion that stress may induce GD but not AIT (35). Our investigation also yielded no evidence linking PTSD to AIT, which consistence with previous study.

The disparity in the relationship between PTSD and GD compared to AIT may be attributed to the distinct pathogenesis of these two diseases, despite sharing some genetic background. GD is the most prevalent cause of persistent hyperthyroidism due to excessive production of thyroid hormones (16), while AIT characterized by ectopic formation of tertiary lymphoid follicles within the thyroid gland and destruction of thyroid follicles (36, 37). AIT involves a Th1-mediated immune response with production of thyroid-specific antibodies leading to hypothyroidism (16, 38). Conversely, GD is characterized by a Th2-mediated immune response with production of stimulating antibodies causing hyperthyroidism (8, 36, 39). Stress has been associated with an increased risk of GD due to its ability to induce excessive cortisol output and suppress immune response (40, 41). Stress induces activation of the hypothalamic-pituitary-adrenal (HPA) axis, eliciting the secretion of glucocorticoids and catecholamines (42). These bioactive molecules exert inhibitory effects on the production of Interleukin 12 (IL-12) by antigen-presenting cells (APCs) and downregulate IL-12 receptor expression on T cells. Concurrently, they upregulate the synthesis of IL-4 and IL-10 by Th2 cells (43), thereby fostering dysregulation and differentiation of Th2 cells, ultimately promoting the progression of humoral immunity, which is associated with the initiation of GD (44). Clinical studies have demonstrated that intrathyroid injection of dexamethasone (IID) can effectively prevent the recurrence of GD by inhibiting peripheral blood Th2 cells (39).

Our study is the inaugural endeavor to explore the causal relationship between PTSD and AITD employing MR Analysis and GWAS-level summary data. This approach adeptly addresses potential confounders and mitigates reverse causation by comprehensively aggregating extensive genetic data. However, it is subject to certain limitations. Firstly, the analysis in this study solely focuses on the relationship between PTSD and AIT and GD due to the availability of datasets in the GWAS database. Therefore, the findings may not be applicable to all types of autoimmune thyroid diseases (AITD). Secondly, it is important to acknowledge that our data exclusively comprises individuals of European ancestry as the GWAS database lacks matched data for Asian and African ancestries. Consequently, caution should be exercised when generalizing the results of this study to other racial or ethnic groups. Thirdly, while this study establishes a causal relationship between PTSD and GD, it does not elucidate the underlying biological mechanisms driving this effect.

In the forthcoming years, it is imperative to establish research centers across various continents and countries to delve into the ramifications of PTSD on individuals suffering from AITD across diverse racial demographics. Moreover, concerted attention should be directed towards delving into the underlying biological pathways that link PTSD with immune-mediated inflammatory conditions, as well as exploring potential correlations between the severity of PTSD and the susceptibility or severity of autoimmune ailments. Additionally, investigating whether effective management of PTSD can mitigate the risks associated with autoimmune thyroid disease is imperative. Notably, PTSD is one of the most preventable mental disorders, as evidenced by controlled clinical trials indicating a significant reduction in PTSD risk through early preventive interventions (2). Hence, the provision of comprehensive social and medical care aimed at delivering stress management strategies and supportive measures to individuals predisposed to PTSD may yield benefits extending beyond the realm of PTSD prevention to encompass potential preventive measures against GD.

## 5 Conclusion

Our study indicate that individuals with PTSD may have an increased likelihood of developing GD, underscoring the importance of further research to comprehend the intricate interplay between PTSD and thyroid disorders. And, facilitating the implementation of stress management strategies and supportive interventions for individuals predisposed to PTSD may holds the potential for yielding multifaceted benefits.

## Data Availability

The original contributions presented in the study are included in the article/**Supplementary Material**. Further inquiries can be directed to the corresponding author.
