# Supplementary material for: Causal effects of post-traumatic stress disorder on autoimmune thyroid disease: insights from mendelian randomization
Source: Front Psychiatry. 2024 Sep 20;15:1417302. doi: 10.3389/fpsyt.2024.1417302 (PMC11449846; doi:10.3389/fpsyt.2024.1417302)
Supplement: Supplementary file 1 [file Table1.docx]

**Supplementary Table S1** Characteristics of instrumental variables for PTSD

|  | **SNP** | **EA** | **OA** | **Samplesize** | **SE** | **β** | **id.exposure** | **EAF** | ***p* value** | **R^2^** | **F - statistic** |
| --- | --- | --- | --- | --- | --- | --- | --- | --- | --- | --- | --- |
| 1 | rs61751012 | A | G | 339,859 | 0.130456 | 0.60627 | finngen_R9_F5_PTSD | 0.00928262 | 3.36E-06 | 0.006760557 | 2313.261708 |
| 2 | rs7530037 | C | T | 339,859 | 0.0341907 | -0.138833 | finngen_R9_F5_PTSD | 0.749598 | 4.90E-05 | 0.007235718 | 2477.032476 |
| 3 | rs112685439 | C | T | 339,859 | 0.071243 | -0.298621 | finngen_R9_F5_PTSD | 0.0554596 | 2.77E-05 | 0.009342604 | 3205.09341 |
| 4 | rs76993046 | C | T | 339,859 | 0.122203 | -0.506649 | finngen_R9_F5_PTSD | 0.0209109 | 3.38E-05 | 0.010510886 | 3610.143914 |
| 5 | rs955822 | T | C | 339,859 | 0.0383737 | -0.161672 | finngen_R9_F5_PTSD | 0.200148 | 2.52E-05 | 0.008368748 | 2868.180781 |
| 6 | rs72788969 | T | C | 339,859 | 0.0442647 | 0.185977 | finngen_R9_F5_PTSD | 0.117263 | 2.65E-05 | 0.007160458 | 2451.082654 |
| 7 | rs72827609 | A | G | 339,859 | 0.133413 | -0.650624 | finngen_R9_F5_PTSD | 0.0199395 | 1.08E-06 | 0.016544639 | 5717.403874 |
| 8 | rs868209 | A | T | 339,859 | 0.0456582 | -0.195814 | finngen_R9_F5_PTSD | 0.146312 | 1.80E-05 | 0.00957848 | 3286.795992 |
| 9 | rs4851628 | T | G | 339,859 | 0.0314334 | 0.130457 | finngen_R9_F5_PTSD | 0.330738 | 3.32E-05 | 0.007534337 | 2580.035978 |
| 10 | rs4849528 | A | C | 339,859 | 0.0419542 | 0.170402 | finngen_R9_F5_PTSD | 0.137931 | 4.87E-05 | 0.006905312 | 2363.136698 |
| 11 | rs35079494 | C | T | 339,859 | 0.0381443 | -0.157812 | finngen_R9_F5_PTSD | 0.204754 | 3.52E-05 | 0.008110431 | 2778.925012 |
| 12 | rs769018 | T | C | 339,859 | 0.0532497 | 0.246554 | finngen_R9_F5_PTSD | 0.900381 | 3.65E-06 | 0.010904923 | 3746.974867 |
| 13 | rs28823579 | T | G | 339,859 | 0.0399014 | -0.177824 | finngen_R9_F5_PTSD | 0.189183 | 8.33E-06 | 0.009700982 | 3329.243579 |
| 14 | rs115056533 | A | G | 339,859 | 0.0991646 | 0.418943 | finngen_R9_F5_PTSD | 0.0182653 | 2.39E-05 | 0.006294494 | 2152.778498 |
| 15 | rs147667696 | T | C | 339,859 | 0.145723 | -0.591326 | finngen_R9_F5_PTSD | 0.0148869 | 4.95E-05 | 0.010255913 | 3521.661539 |
| 16 | rs35467279 | A | C | 339,859 | 0.0320289 | 0.146247 | finngen_R9_F5_PTSD | 0.656553 | 4.97E-06 | 0.009645693 | 3310.084419 |
| 17 | rs2637766 | G | A | 339,859 | 0.0357007 | -0.147341 | finngen_R9_F5_PTSD | 0.246966 | 3.67E-05 | 0.008074748 | 2766.599205 |
| 18 | rs62253086 | G | T | 339,859 | 0.0763446 | -0.311856 | finngen_R9_F5_PTSD | 0.0496284 | 4.41E-05 | 0.009174067 | 3146.739433 |
| 19 | rs144580248 | C | T | 339,859 | 0.0488899 | 0.20079 | finngen_R9_F5_PTSD | 0.092917 | 4.01E-05 | 0.006796047 | 2325.488211 |
| 20 | rs142692233 | A | G | 339,859 | 0.0810456 | 0.331106 | finngen_R9_F5_PTSD | 0.0295568 | 4.40E-05 | 0.006289145 | 2150.937627 |
| 21 | rs74549297 | G | A | 339,859 | 0.113152 | 0.472855 | finngen_R9_F5_PTSD | 0.0127224 | 2.93E-05 | 0.005616869 | 1919.715042 |
| 22 | rs6855181 | C | T | 339,859 | 0.030474 | -0.125118 | finngen_R9_F5_PTSD | 0.607499 | 4.03E-05 | 0.007465449 | 2556.268706 |
| 23 | rs142659084 | C | T | 339,859 | 0.0752442 | 0.316394 | finngen_R9_F5_PTSD | 0.035189 | 2.61E-05 | 0.006797288 | 2325.915688 |
| 24 | rs12509070 | G | C | 339,859 | 0.0317984 | -0.128982 | finngen_R9_F5_PTSD | 0.351857 | 4.99E-05 | 0.007587964 | 2598.540179 |
| 25 | rs79635473 | A | G | 339,859 | 0.0612903 | 0.272105 | finngen_R9_F5_PTSD | 0.0544446 | 9.01E-06 | 0.007623332 | 2610.745301 |
| 26 | rs907238 | C | T | 339,859 | 0.0676802 | 0.326466 | finngen_R9_F5_PTSD | 0.936571 | 1.41E-06 | 0.012662938 | 4358.783141 |
| 27 | rs6536404 | A | G | 339,859 | 0.0307888 | -0.131783 | finngen_R9_F5_PTSD | 0.632922 | 1.87E-05 | 0.008069699 | 2764.855167 |
| 28 | rs76506460 | T | C | 339,859 | 0.115024 | 0.48866 | finngen_R9_F5_PTSD | 0.0123811 | 2.15E-05 | 0.005839722 | 1996.328531 |
| 29 | rs10060769 | T | C | 339,859 | 0.0319749 | 0.14212 | finngen_R9_F5_PTSD | 0.305865 | 8.80E-06 | 0.00857658 | 2940.025963 |
| 30 | rs200299868 | G | A | 339,859 | 0.0556654 | -0.230529 | finngen_R9_F5_PTSD | 0.929926 | 3.45E-05 | 0.006926063 | 2370.287701 |
| 31 | rs73142278 | T | C | 339,859 | 0.131946 | 0.602307 | finngen_R9_F5_PTSD | 0.00920632 | 5.00E-06 | 0.006618127 | 2264.201634 |
| 32 | rs4620012 | G | A | 339,859 | 0.0326539 | -0.138896 | finngen_R9_F5_PTSD | 0.316793 | 2.10E-05 | 0.008350978 | 2862.039275 |
| 33 | rs74863973 | C | T | 339,859 | 0.0398096 | -0.169312 | finngen_R9_F5_PTSD | 0.18389 | 2.11E-05 | 0.008604235 | 2949.588612 |
| 34 | rs80038448 | T | C | 339,859 | 0.0457048 | 0.213158 | finngen_R9_F5_PTSD | 0.108838 | 3.10E-06 | 0.008813948 | 3022.118704 |
| 35 | rs56066057 | G | T | 339,859 | 0.0310477 | 0.127332 | finngen_R9_F5_PTSD | 0.358165 | 4.11E-05 | 0.007454382 | 2552.450922 |
| 36 | rs147189267 | T | C | 339,859 | 0.113527 | 0.465528 | finngen_R9_F5_PTSD | 0.013458 | 4.12E-05 | 0.005754634 | 1967.072459 |
| 37 | rs116174051 | C | T | 339,859 | 0.134479 | 0.558638 | finngen_R9_F5_PTSD | 0.00865994 | 3.27E-05 | 0.005358318 | 1830.872274 |
| 38 | rs9463851 | G | A | 339,859 | 0.0402399 | -0.170269 | finngen_R9_F5_PTSD | 0.183282 | 2.32E-05 | 0.008679468 | 2975.60464 |
| 39 | rs12193488 | G | A | 339,859 | 0.0421757 | 0.172597 | finngen_R9_F5_PTSD | 0.135621 | 4.27E-05 | 0.006984376 | 2390.384525 |
| 40 | rs137910645 | T | C | 339,859 | 0.128409 | 0.53158 | finngen_R9_F5_PTSD | 0.0104076 | 3.48E-05 | 0.005820686 | 1989.782936 |
| 41 | rs112875421 | C | G | 339,859 | 0.0575258 | 0.242591 | finngen_R9_F5_PTSD | 0.0678203 | 2.47E-05 | 0.007441127 | 2547.87816 |
| 42 | rs3857844 | G | C | 339,859 | 0.0379508 | 0.154322 | finngen_R9_F5_PTSD | 0.17961 | 4.78E-05 | 0.007018375 | 2402.102637 |
| 43 | rs147761221 | A | G | 339,859 | 0.100985 | -0.437439 | finngen_R9_F5_PTSD | 0.0283436 | 1.48E-05 | 0.010539809 | 3620.183754 |
| 44 | rs17618440 | G | T | 339,859 | 0.2796 | -1.13642 | finngen_R9_F5_PTSD | 0.00586284 | 4.81E-05 | 0.015054353 | 5194.527339 |
| 45 | rs117042680 | C | A | 339,859 | 0.13691 | 0.577638 | finngen_R9_F5_PTSD | 0.00877628 | 2.45E-05 | 0.005805287 | 1984.487802 |
| 46 | rs147901527 | A | G | 339,859 | 0.0700035 | -0.289142 | finngen_R9_F5_PTSD | 0.0561424 | 3.62E-05 | 0.008860328 | 3038.163683 |
| 47 | rs4076019 | G | A | 339,859 | 0.0300606 | -0.126283 | finngen_R9_F5_PTSD | 0.515503 | 2.66E-05 | 0.007966032 | 2729.051571 |
| 48 | rs118062537 | C | T | 339,859 | 0.285985 | -1.19971 | finngen_R9_F5_PTSD | 0.00597691 | 2.73E-05 | 0.017102348 | 5913.487243 |
| 49 | rs139734635 | C | T | 339,859 | 0.194243 | 0.804768 | finngen_R9_F5_PTSD | 0.0036 | 3.43E-05 | 0.004646304 | 1586.450039 |
| 50 | rs7867845 | G | C | 339,859 | 0.045819 | -0.199292 | finngen_R9_F5_PTSD | 0.132095 | 1.36E-05 | 0.009106852 | 3123.472529 |
| 51 | rs3956254 | T | C | 339,859 | 0.0400637 | -0.169196 | finngen_R9_F5_PTSD | 0.18314 | 2.41E-05 | 0.008565269 | 2936.115324 |
| 52 | rs117766016 | A | G | 339,859 | 0.0487789 | 0.21026 | finngen_R9_F5_PTSD | 0.0921022 | 1.63E-05 | 0.007393504 | 2531.45021 |
| 53 | rs7918981 | A | G | 339,859 | 0.0722265 | 0.327741 | finngen_R9_F5_PTSD | 0.0381038 | 5.69E-06 | 0.007873866 | 2697.226219 |
| 54 | rs187042189 | T | C | 339,859 | 0.0706974 | 0.290682 | finngen_R9_F5_PTSD | 0.038795 | 3.93E-05 | 0.006301705 | 2155.260289 |
| 55 | rs9971308 | G | A | 339,859 | 0.0372816 | 0.17341 | finngen_R9_F5_PTSD | 0.785755 | 3.30E-06 | 0.010124559 | 3476.09623 |
| 56 | rs6585536 | C | T | 339,859 | 0.0309489 | -0.131702 | finngen_R9_F5_PTSD | 0.641574 | 2.09E-05 | 0.007977393 | 2732.974927 |
| 57 | rs12418369 | A | G | 339,859 | 0.0769038 | 0.316797 | finngen_R9_F5_PTSD | 0.0344927 | 3.80E-05 | 0.006684591 | 2287.093482 |
| 58 | rs2033975 | T | C | 339,859 | 0.145161 | -0.60286 | finngen_R9_F5_PTSD | 0.0172161 | 3.28E-05 | 0.012298602 | 4231.811309 |
| 59 | rs139700175 | T | C | 339,859 | 0.224792 | -0.952387 | finngen_R9_F5_PTSD | 0.00799281 | 2.27E-05 | 0.01438372 | 4959.747571 |
| 60 | rs2510737 | C | G | 339,859 | 0.0309694 | 0.12882 | finngen_R9_F5_PTSD | 0.367874 | 3.19E-05 | 0.007717903 | 2643.384855 |
| 61 | rs4548693 | C | A | 339,859 | 0.0337578 | -0.155318 | finngen_R9_F5_PTSD | 0.75159 | 4.21E-06 | 0.009007902 | 3089.225924 |
| 62 | rs75758664 | A | C | 339,859 | 0.187507 | 0.781302 | finngen_R9_F5_PTSD | 0.00432206 | 3.09E-05 | 0.005253848 | 1794.98778 |
| 63 | rs145086244 | A | G | 339,859 | 0.207972 | 0.883138 | finngen_R9_F5_PTSD | 0.00337917 | 2.17E-05 | 0.005253239 | 1794.778368 |
| 64 | rs1536900 | C | T | 339,859 | 0.030208 | -0.134555 | finngen_R9_F5_PTSD | 0.586919 | 8.42E-06 | 0.00877896 | 3010.015784 |
| 65 | rs17636116 | T | C | 339,859 | 0.0852231 | -0.350007 | finngen_R9_F5_PTSD | 0.0397059 | 4.01E-05 | 0.009342062 | 3204.90571 |
| 66 | rs148649817 | A | G | 339,859 | 0.0874778 | -0.374444 | finngen_R9_F5_PTSD | 0.0399309 | 1.87E-05 | 0.01075017 | 3693.223359 |
| 67 | rs2225230 | C | T | 339,859 | 0.0387108 | -0.16423 | finngen_R9_F5_PTSD | 0.829635 | 2.21E-05 | 0.007624343 | 2611.094208 |
| 68 | rs76376367 | G | T | 339,859 | 0.158696 | 0.652224 | finngen_R9_F5_PTSD | 0.00653177 | 3.96E-05 | 0.005520881 | 1886.726567 |
| 69 | rs8004419 | A | T | 339,859 | 0.0318584 | 0.137268 | finngen_R9_F5_PTSD | 0.65335 | 1.64E-05 | 0.008535043 | 2925.664745 |
| 70 | rs57729369 | G | A | 339,859 | 0.0634721 | 0.267582 | finngen_R9_F5_PTSD | 0.0526173 | 2.49E-05 | 0.007138349 | 2443.460251 |
| 71 | rs113058811 | T | C | 339,859 | 0.0906465 | -0.408074 | finngen_R9_F5_PTSD | 0.037075 | 6.74E-06 | 0.011889989 | 4089.520485 |
| 72 | rs79508047 | G | A | 339,859 | 0.0568692 | -0.254873 | finngen_R9_F5_PTSD | 0.085999 | 7.40E-06 | 0.010212163 | 3506.483796 |
| 73 | rs531586818 | G | C | 339,859 | 0.253964 | -1.07311 | finngen_R9_F5_PTSD | 0.00660351 | 2.38E-05 | 0.015108312 | 5213.431693 |
| 74 | rs79817593 | T | C | 339,859 | 0.102826 | -0.41989 | finngen_R9_F5_PTSD | 0.0290894 | 4.44E-05 | 0.009958985 | 3418.677217 |
| 75 | rs2081249 | T | C | 339,859 | 0.0471167 | -0.191369 | finngen_R9_F5_PTSD | 0.899531 | 4.87E-05 | 0.006619442 | 2264.654468 |
| 76 | rs11864961 | A | G | 339,859 | 0.0402499 | -0.180524 | finngen_R9_F5_PTSD | 0.178562 | 7.29E-06 | 0.009560128 | 3280.437947 |
| 77 | rs2993355 | G | A | 339,859 | 0.031509 | -0.129075 | finngen_R9_F5_PTSD | 0.406487 | 4.20E-05 | 0.008038799 | 2754.182367 |
| 78 | rs11868787 | A | G | 339,859 | 0.0384711 | -0.157748 | finngen_R9_F5_PTSD | 0.207488 | 4.12E-05 | 0.008183829 | 2804.28136 |
| 79 | rs17213068 | G | C | 339,859 | 0.0548639 | -0.252314 | finngen_R9_F5_PTSD | 0.0957186 | 4.25E-06 | 0.011020788 | 3787.230038 |
| 80 | rs6504128 | C | A | 339,859 | 0.0806945 | -0.330515 | finngen_R9_F5_PTSD | 0.969168 | 4.21E-05 | 0.006528495 | 2233.335206 |
| 81 | rs7502307 | G | C | 339,859 | 0.0338021 | 0.154206 | finngen_R9_F5_PTSD | 0.250873 | 5.07E-06 | 0.008938032 | 3065.048297 |
| 82 | rs7503604 | A | C | 339,859 | 0.0301801 | -0.124948 | finngen_R9_F5_PTSD | 0.48511 | 3.47E-05 | 0.007799079 | 2671.405965 |
| 83 | rs150725653 | C | A | 339,859 | 0.103859 | -0.444805 | finngen_R9_F5_PTSD | 0.0294041 | 1.85E-05 | 0.011293165 | 3881.899995 |
| 84 | rs10468956 | T | C | 339,859 | 0.0418477 | 0.18134 | finngen_R9_F5_PTSD | 0.13207 | 1.47E-05 | 0.007538867 | 2581.599142 |
| 85 | rs11665300 | A | G | 339,859 | 0.0637197 | 0.274787 | finngen_R9_F5_PTSD | 0.0504604 | 1.61E-05 | 0.007235793 | 2477.058367 |
| 86 | rs12959593 | T | C | 339,859 | 0.0307495 | 0.131096 | finngen_R9_F5_PTSD | 0.59645 | 2.01E-05 | 0.008273329 | 2835.205207 |
| 87 | rs138295459 | T | C | 339,859 | 0.231808 | -0.982456 | finngen_R9_F5_PTSD | 0.00828451 | 2.25E-05 | 0.015860254 | 5477.086314 |
| 88 | rs12460276 | A | G | 339,859 | 0.0787991 | -0.376088 | finngen_R9_F5_PTSD | 0.0472013 | 1.82E-06 | 0.012722254 | 4379.463752 |
| 89 | rs927170 | G | T | 339,859 | 0.0505011 | 0.21023 | finngen_R9_F5_PTSD | 0.0865346 | 3.14E-05 | 0.006987169 | 2391.347157 |
| 90 | rs6094353 | G | A | 339,859 | 0.0603975 | -0.268998 | finngen_R9_F5_PTSD | 0.0800681 | 8.44E-06 | 0.010659659 | 3661.79309 |
| 91 | rs6021953 | A | G | 339,859 | 0.0346004 | -0.168434 | finngen_R9_F5_PTSD | 0.268939 | 1.13E-06 | 0.011155702 | 3834.115827 |
| 92 | rs367001 | T | G | 339,859 | 0.0301593 | -0.125527 | finngen_R9_F5_PTSD | 0.477535 | 3.15E-05 | 0.007862609 | 2693.339544 |
| 93 | rs138861164 | A | C | 339,859 | 0.222522 | -0.959288 | finngen_R9_F5_PTSD | 0.00835338 | 1.63E-05 | 0.015245694 | 5261.572079 |

SNP, single nucleotide polymorphism; EA, effect allele; OA, other allele; EAF, effect allele frequency; SE, standard error; PTSD, post-traumatic stress disorder.

**Supplementary Table S2.1** Information of identified SNPs in exposure (PTSD) and outcomes (AIT).

|  |  | | | **Exposure (PTSD)** | | |  | **Outcome (AIT)** | | | | |
| --- | --- | --- | --- | --- | --- | --- | --- | --- | --- | --- | --- | --- |
|  | **SNP** | **EA** | **OA** | **β** | **SE** | ***p* value** |  | **Case** | **Control** | **β** | **SE** | ***p* value** |
| 1 | rs10060769 | T | C | 0.14212 | 0.0319749 | 8.80E-06 |  | 489 | 320,703 | -0.075903 | 0.0695662 | 0.275233 |
| 2 | rs10468956 | T | C | 0.18134 | 0.0418477 | 1.47E-05 |  | 489 | 320,703 | 0.126894 | 0.0950202 | 0.181732 |
| 3 | rs112685439 | C | T | -0.298621 | 0.071243 | 2.77E-05 |  | 489 | 320,703 | -0.109125 | 0.141706 | 0.441253 |
| 4 | rs112875421 | C | G | 0.242591 | 0.0575258 | 2.47E-05 |  | 489 | 320,703 | 0.00165943 | 0.131797 | 0.989954 |
| 5 | rs113058811 | T | C | -0.408074 | 0.0906465 | 6.74E-06 |  | 489 | 320,703 | 0.00256833 | 0.168605 | 0.987846 |
| 6 | rs115056533 | A | G | 0.418943 | 0.0991646 | 2.39E-05 |  | 489 | 320,703 | -0.217847 | 0.243835 | 0.371632 |
| 7 | rs116174051 | C | T | 0.558638 | 0.134479 | 3.27E-05 |  | 489 | 320,703 | -0.045885 | 0.347022 | 0.894806 |
| 8 | rs11665300 | A | G | 0.274787 | 0.0637197 | 1.61E-05 |  | 489 | 320,703 | 0.0324127 | 0.148575 | 0.827306 |
| 9 | rs117042680 | C | A | 0.577638 | 0.13691 | 2.45E-05 |  | 489 | 320,703 | -0.0431861 | 0.338561 | 0.898499 |
| 10 | rs117766016 | A | G | 0.21026 | 0.0487789 | 1.63E-05 |  | 489 | 320,703 | -0.0770427 | 0.111347 | 0.48899 |
| 11 | rs118062537 | C | T | -1.19971 | 0.285985 | 2.73E-05 |  | 489 | 320,703 | 0.446361 | 0.403069 | 0.268117 |
| 12 | rs11864961 | A | G | -0.180524 | 0.0402499 | 7.29E-06 |  | 489 | 320,703 | -0.0125762 | 0.0842902 | 0.881395 |
| 13 | rs11868787 | A | G | -0.157748 | 0.0384711 | 4.12E-05 |  | 489 | 320,703 | 0.187317 | 0.0798442 | 0.0189745 |
| 14 | rs12193488 | G | A | 0.172597 | 0.0421757 | 4.27E-05 |  | 489 | 320,703 | 0.0808774 | 0.0941573 | 0.390362 |
| 15 | rs12418369 | A | G | 0.316797 | 0.0769038 | 3.80E-05 |  | 489 | 320,703 | 0.0306975 | 0.178323 | 0.863323 |
| 16 | rs12460276 | A | G | -0.376088 | 0.0787991 | 1.82E-06 |  | 489 | 320,703 | -1.86E-05 | 0.149255 | 0.9999 |
| 17 | rs12509070 | G | C | -0.128982 | 0.0317984 | 4.99E-05 |  | 489 | 320,703 | -0.00649972 | 0.067346 | 0.923114 |
| 18 | rs12959593 | T | C | 0.131096 | 0.0307495 | 2.01E-05 |  | 489 | 320,703 | 0.0760508 | 0.0657455 | 0.247377 |
| 19 | rs137910645 | T | C | 0.53158 | 0.128409 | 3.48E-05 |  | 489 | 320,703 | -0.230193 | 0.32384 | 0.477194 |
| 20 | rs138295459 | T | C | -0.982456 | 0.231808 | 2.25E-05 |  | 489 | 320,703 | -0.124264 | 0.355801 | 0.726901 |
| 21 | rs138861164 | A | C | -0.959288 | 0.222522 | 1.63E-05 |  | 489 | 320,703 | 0.193925 | 0.349516 | 0.579005 |
| 22 | rs139700175 | T | C | -0.952387 | 0.224792 | 2.27E-05 |  | 489 | 320,703 | -0.140707 | 0.352688 | 0.689927 |
| 23 | rs139734635 | C | T | 0.804768 | 0.194243 | 3.43E-05 |  | 489 | 320,703 | -0.634874 | 0.543411 | 0.242681 |
| 24 | rs142659084 | C | T | 0.316394 | 0.0752442 | 2.61E-05 |  | 489 | 320,703 | -0.116338 | 0.173082 | 0.501484 |
| 25 | rs142692233 | A | G | 0.331106 | 0.0810456 | 4.40E-05 |  | 489 | 320,703 | -0.161437 | 0.187899 | 0.390246 |
| 26 | rs144580248 | C | T | 0.20079 | 0.0488899 | 4.01E-05 |  | 489 | 320,703 | -0.0224891 | 0.111371 | 0.839972 |
| 27 | rs145086244 | A | G | 0.883138 | 0.207972 | 2.17E-05 |  | 489 | 320,703 | -0.858644 | 0.572218 | 0.133471 |
| 28 | rs147189267 | T | C | 0.465528 | 0.113527 | 4.12E-05 |  | 489 | 320,703 | -0.247109 | 0.291234 | 0.396165 |
| 29 | rs147667696 | T | C | -0.591326 | 0.145723 | 4.95E-05 |  | 489 | 320,703 | -0.265969 | 0.275464 | 0.33428 |
| 30 | rs147761221 | A | G | -0.437439 | 0.100985 | 1.48E-05 |  | 489 | 320,703 | 0.239537 | 0.194593 | 0.218336 |
| 31 | rs147901527 | A | G | -0.289142 | 0.0700035 | 3.62E-05 |  | 489 | 320,703 | -0.233449 | 0.140587 | 0.0968077 |
| 32 | rs148649817 | A | G | -0.374444 | 0.0874778 | 1.87E-05 |  | 489 | 320,703 | -0.0570276 | 0.163099 | 0.726601 |
| 33 | rs150725653 | C | A | -0.444805 | 0.103859 | 1.85E-05 |  | 489 | 320,703 | 0.163905 | 0.190269 | 0.388998 |
| 34 | rs1536900 | C | T | -0.134555 | 0.030208 | 8.42E-06 |  | 489 | 320,703 | 0.104849 | 0.0652814 | 0.108251 |
| 35 | rs17213068 | G | C | -0.252314 | 0.0548639 | 4.25E-06 |  | 489 | 320,703 | 0.00498824 | 0.108788 | 0.963427 |
| 36 | rs17618440 | G | T | -1.13642 | 0.2796 | 4.81E-05 |  | 489 | 320,703 | 0.250406 | 0.400107 | 0.531415 |
| 37 | rs17636116 | T | C | -0.350007 | 0.0852231 | 4.01E-05 |  | 489 | 320,703 | 0.277038 | 0.167525 | 0.0981861 |
| 38 | rs187042189 | T | C | 0.290682 | 0.0706974 | 3.93E-05 |  | 489 | 320,703 | 0.0712585 | 0.170842 | 0.676605 |
| 39 | rs200299868 | G | A | -0.230529 | 0.0556654 | 3.45E-05 |  | 489 | 320,703 | -0.0481618 | 0.124639 | 0.699194 |
| 40 | rs2033975 | T | C | -0.60286 | 0.145161 | 3.28E-05 |  | 489 | 320,703 | -0.257562 | 0.243137 | 0.28945 |
| 41 | rs2081249 | T | C | -0.191369 | 0.0471167 | 4.87E-05 |  | 489 | 320,703 | 0.11538 | 0.107873 | 0.284802 |
| 42 | rs2225230 | C | T | -0.16423 | 0.0387108 | 2.21E-05 |  | 489 | 320,703 | 0.0736388 | 0.0867028 | 0.395701 |
| 43 | rs2510737 | C | G | 0.12882 | 0.0309694 | 3.19E-05 |  | 489 | 320,703 | 0.0397731 | 0.0674027 | 0.555135 |
| 44 | rs2637766 | G | A | -0.147341 | 0.0357007 | 3.67E-05 |  | 489 | 320,703 | 0.0918441 | 0.0740499 | 0.214864 |
| 45 | rs28823579 | T | G | -0.177824 | 0.0399014 | 8.33E-06 |  | 489 | 320,703 | 0.0779247 | 0.081833 | 0.340975 |
| 46 | rs2993355 | G | A | -0.129075 | 0.031509 | 4.20E-05 |  | 489 | 320,703 | 0.0260384 | 0.067483 | 0.699607 |
| 47 | rs35079494 | C | T | -0.157812 | 0.0381443 | 3.52E-05 |  | 489 | 320,703 | 0.0182177 | 0.0794515 | 0.818641 |
| 48 | rs35467279 | A | C | 0.146247 | 0.0320289 | 4.97E-06 |  | 489 | 320,703 | 0.0884212 | 0.0677489 | 0.191849 |
| 49 | rs367001 | T | G | -0.125527 | 0.0301593 | 3.15E-05 |  | 489 | 320,703 | 0.0074056 | 0.0644763 | 0.908558 |
| 50 | rs3857844 | G | C | 0.154322 | 0.0379508 | 4.78E-05 |  | 489 | 320,703 | 0.0208962 | 0.0835029 | 0.802398 |
| 51 | rs3956254 | T | C | -0.169196 | 0.0400637 | 2.41E-05 |  | 489 | 320,703 | 0.0226364 | 0.0839475 | 0.78743 |
| 52 | rs4076019 | G | A | -0.126283 | 0.0300606 | 2.66E-05 |  | 489 | 320,703 | 0.0308756 | 0.0647614 | 0.633534 |
| 53 | rs4548693 | C | A | -0.155318 | 0.0337578 | 4.21E-06 |  | 489 | 320,703 | -0.0863919 | 0.0741692 | 0.244102 |
| 54 | rs4620012 | G | A | -0.138896 | 0.0326539 | 2.10E-05 |  | 489 | 320,703 | 0.0626757 | 0.0690497 | 0.364042 |
| 55 | rs4849528 | A | C | 0.170402 | 0.0419542 | 4.87E-05 |  | 489 | 320,703 | -0.10301 | 0.0949872 | 0.278158 |
| 56 | rs4851628 | T | G | 0.130457 | 0.0314334 | 3.32E-05 |  | 489 | 320,703 | 0.0307548 | 0.0683321 | 0.652654 |
| 57 | rs531586818 | G | C | -1.07311 | 0.253964 | 2.38E-05 |  | 489 | 320,703 | -0.284202 | 0.386552 | 0.462203 |
| 58 | rs56066057 | G | T | 0.127332 | 0.0310477 | 4.11E-05 |  | 489 | 320,703 | 0.155612 | 0.0673591 | 0.0208776 |
| 59 | rs57729369 | G | A | 0.267582 | 0.0634721 | 2.49E-05 |  | 489 | 320,703 | -0.351572 | 0.148532 | 0.0179341 |
| 60 | rs6021953 | A | G | -0.168434 | 0.0346004 | 1.13E-06 |  | 489 | 320,703 | 0.0058453 | 0.0723374 | 0.935596 |
| 61 | rs6094353 | G | A | -0.268998 | 0.0603975 | 8.44E-06 |  | 489 | 320,703 | 0.0296534 | 0.117437 | 0.800651 |
| 62 | rs61751012 | A | G | 0.60627 | 0.130456 | 3.36E-06 |  | 489 | 320,703 | 0.164868 | 0.344297 | 0.632044 |
| 63 | rs62253086 | G | T | -0.311856 | 0.0763446 | 4.41E-05 |  | 489 | 320,703 | -0.0670625 | 0.147481 | 0.64931 |
| 64 | rs6504128 | C | A | -0.330515 | 0.0806945 | 4.21E-05 |  | 489 | 320,703 | 0.215627 | 0.190781 | 0.258378 |
| 65 | rs6536404 | A | G | -0.131783 | 0.0307888 | 1.87E-05 |  | 489 | 320,703 | 0.0805242 | 0.0667846 | 0.227922 |
| 66 | rs6585536 | C | T | -0.131702 | 0.0309489 | 2.09E-05 |  | 489 | 320,703 | -0.041787 | 0.067128 | 0.533615 |
| 67 | rs6855181 | C | T | -0.125118 | 0.030474 | 4.03E-05 |  | 489 | 320,703 | -0.0324814 | 0.065736 | 0.621222 |
| 68 | rs72788969 | T | C | 0.185977 | 0.0442647 | 2.65E-05 |  | 489 | 320,703 | -0.0805494 | 0.101478 | 0.427335 |
| 69 | rs72827609 | A | G | -0.650624 | 0.133413 | 1.08E-06 |  | 489 | 320,703 | -0.364469 | 0.227521 | 0.109175 |
| 70 | rs73142278 | T | C | 0.602307 | 0.131946 | 5.00E-06 |  | 489 | 320,703 | -0.576488 | 0.34206 | 0.0919221 |
| 71 | rs74549297 | G | A | 0.472855 | 0.113152 | 2.93E-05 |  | 489 | 320,703 | 0.127537 | 0.286877 | 0.65663 |
| 72 | rs74863973 | C | T | -0.169312 | 0.0398096 | 2.11E-05 |  | 489 | 320,703 | -0.163879 | 0.083357 | 0.0492992 |
| 73 | rs7502307 | G | C | 0.154206 | 0.0338021 | 5.07E-06 |  | 489 | 320,703 | 0.0207658 | 0.0741432 | 0.779419 |
| 74 | rs7503604 | A | C | -0.124948 | 0.0301801 | 3.47E-05 |  | 489 | 320,703 | 0.0854986 | 0.0647429 | 0.186639 |
| 75 | rs7530037 | C | T | -0.138833 | 0.0341907 | 4.90E-05 |  | 489 | 320,703 | -0.052634 | 0.0742938 | 0.478661 |
| 76 | rs75758664 | A | C | 0.781302 | 0.187507 | 3.09E-05 |  | 489 | 320,703 | -0.315147 | 0.491425 | 0.521333 |
| 77 | rs76376367 | G | T | 0.652224 | 0.158696 | 3.96E-05 |  | 489 | 320,703 | -0.404819 | 0.396197 | 0.306894 |
| 78 | rs76506460 | T | C | 0.48866 | 0.115024 | 2.15E-05 |  | 489 | 320,703 | -0.284075 | 0.284771 | 0.318495 |
| 79 | rs769018 | T | C | 0.246554 | 0.0532497 | 3.65E-06 |  | 489 | 320,703 | -0.12179 | 0.107346 | 0.256564 |
| 80 | rs76993046 | C | T | -0.506649 | 0.122203 | 3.38E-05 |  | 489 | 320,703 | 0.283959 | 0.223701 | 0.204309 |
| 81 | rs7867845 | G | C | -0.199292 | 0.045819 | 1.36E-05 |  | 489 | 320,703 | 0.026251 | 0.0954866 | 0.783379 |
| 82 | rs7918981 | A | G | 0.327741 | 0.0722265 | 5.69E-06 |  | 489 | 320,703 | 0.308114 | 0.169679 | 0.0693905 |
| 83 | rs79508047 | G | A | -0.254873 | 0.0568692 | 7.40E-06 |  | 489 | 320,703 | -0.198454 | 0.115952 | 0.0869861 |
| 84 | rs79635473 | A | G | 0.272105 | 0.0612903 | 9.01E-06 |  | 489 | 320,703 | -0.105033 | 0.140047 | 0.453263 |
| 85 | rs79817593 | T | C | -0.41989 | 0.102826 | 4.44E-05 |  | 489 | 320,703 | 0.174677 | 0.195462 | 0.371502 |
| 86 | rs80038448 | T | C | 0.213158 | 0.0457048 | 3.10E-06 |  | 489 | 320,703 | 0.0436314 | 0.102286 | 0.6697 |
| 87 | rs8004419 | A | T | 0.137268 | 0.0318584 | 1.64E-05 |  | 489 | 320,703 | 0.017355 | 0.0674727 | 0.797013 |
| 88 | rs868209 | A | T | -0.195814 | 0.0456582 | 1.80E-05 |  | 489 | 320,703 | 0.109999 | 0.0922767 | 0.233237 |
| 89 | rs907238 | C | T | 0.326466 | 0.0676802 | 1.41E-06 |  | 489 | 320,703 | -0.158573 | 0.131071 | 0.226347 |
| 90 | rs927170 | G | T | 0.21023 | 0.0505011 | 3.14E-05 |  | 489 | 320,703 | -0.237511 | 0.113575 | 0.0365073 |
| 91 | rs9463851 | G | A | -0.170269 | 0.0402399 | 2.32E-05 |  | 489 | 320,703 | -0.0236328 | 0.0831414 | 0.77622 |
| 92 | rs955822 | T | C | -0.161672 | 0.0383737 | 2.52E-05 |  | 489 | 320,703 | -0.120212 | 0.080556 | 0.135626 |
| 93 | rs9971308 | G | A | 0.17341 | 0.0372816 | 3.30E-06 |  | 489 | 320,703 | -0.128572 | 0.0782092 | 0.100186 |

SNP, single nucleotide polymorphism; EA, effect allele; OA, other allele; SE, standard error; PTSD, post-traumatic stress disorder; AIT, Autoimmune thyroiditis.

**Supplementary Table S2.2** Information of identified SNPs in exposure (PTSD) and outcomes (GD).

|  |  | | | **Exposure (PTSD)** | | |  | **Outcome (GD)** | | | | |
| --- | --- | --- | --- | --- | --- | --- | --- | --- | --- | --- | --- | --- |
|  | **SNP** | **EA** | **OA** | **β** | **SE** | ***p* value** |  | **Case** | **Control** | **β** | **SE** | ***p* value** |
| 1 | rs10060769 | T | C | 0.14212 | 0.0319749 | 8.80E-06 |  | 2,836 | 374,441 | 0.0382171 | 0.0291114 | 0.189255 |
| 2 | rs10468956 | T | C | 0.18134 | 0.0418477 | 1.47E-05 |  | 2,836 | 374,441 | 0.0463928 | 0.0397668 | 0.243363 |
| 3 | rs112685439 | C | T | -0.298621 | 0.071243 | 2.77E-05 |  | 2,836 | 374,441 | -0.0310294 | 0.0590654 | 0.599347 |
| 4 | rs112875421 | C | G | 0.242591 | 0.0575258 | 2.47E-05 |  | 2,836 | 374,441 | 0.130155 | 0.055045 | 0.0180534 |
| 5 | rs113058811 | T | C | -0.408074 | 0.0906465 | 6.74E-06 |  | 2,836 | 374,441 | 0.0432695 | 0.0705137 | 0.539458 |
| 6 | rs115056533 | A | G | 0.418943 | 0.0991646 | 2.39E-05 |  | 2,836 | 374,441 | -0.0559264 | 0.101645 | 0.582173 |
| 7 | rs116174051 | C | T | 0.558638 | 0.134479 | 3.27E-05 |  | 2,836 | 374,441 | 0.0957926 | 0.146663 | 0.51366 |
| 8 | rs11665300 | A | G | 0.274787 | 0.0637197 | 1.61E-05 |  | 2,836 | 374,441 | 0.101502 | 0.0619726 | 0.101453 |
| 9 | rs117042680 | C | A | 0.577638 | 0.13691 | 2.45E-05 |  | 2,836 | 374,441 | 0.20617 | 0.142801 | 0.148807 |
| 10 | rs117766016 | A | G | 0.21026 | 0.0487789 | 1.63E-05 |  | 2,836 | 374,441 | -0.0523868 | 0.0465 | 0.259913 |
| 11 | rs118062537 | C | T | -1.19971 | 0.285985 | 2.73E-05 |  | 2,836 | 374,441 | 0.173055 | 0.169137 | 0.306231 |
| 12 | rs11864961 | A | G | -0.180524 | 0.0402499 | 7.29E-06 |  | 2,836 | 374,441 | 0.029171 | 0.0352599 | 0.408058 |
| 13 | rs11868787 | A | G | -0.157748 | 0.0384711 | 4.12E-05 |  | 2,836 | 374,441 | -0.0108659 | 0.0331824 | 0.743319 |
| 14 | rs12193488 | G | A | 0.172597 | 0.0421757 | 4.27E-05 |  | 2,836 | 374,441 | -0.0384834 | 0.0390369 | 0.324222 |
| 15 | rs12418369 | A | G | 0.316797 | 0.0769038 | 3.80E-05 |  | 2,836 | 374,441 | -0.0589587 | 0.0739728 | 0.425432 |
| 16 | rs12460276 | A | G | -0.376088 | 0.0787991 | 1.82E-06 |  | 2,836 | 374,441 | 0.0399174 | 0.0623689 | 0.522159 |
| 17 | rs12509070 | G | C | -0.128982 | 0.0317984 | 4.99E-05 |  | 2,836 | 374,441 | 0.007108 | 0.0280866 | 0.800211 |
| 18 | rs12959593 | T | C | 0.131096 | 0.0307495 | 2.01E-05 |  | 2,836 | 374,441 | -0.00407908 | 0.0274451 | 0.881848 |
| 19 | rs137910645 | T | C | 0.53158 | 0.128409 | 3.48E-05 |  | 2,836 | 374,441 | 0.0478633 | 0.13237 | 0.71766 |
| 20 | rs138295459 | T | C | -0.982456 | 0.231808 | 2.25E-05 |  | 2,836 | 374,441 | 0.0385665 | 0.150419 | 0.797647 |
| 21 | rs138861164 | A | C | -0.959288 | 0.222522 | 1.63E-05 |  | 2,836 | 374,441 | 0.168157 | 0.145705 | 0.248463 |
| 22 | rs139700175 | T | C | -0.952387 | 0.224792 | 2.27E-05 |  | 2,836 | 374,441 | 0.00254242 | 0.150419 | 0.986515 |
| 23 | rs139734635 | C | T | 0.804768 | 0.194243 | 3.43E-05 |  | 2,836 | 374,441 | -0.0735393 | 0.231015 | 0.750233 |
| 24 | rs142659084 | C | T | 0.316394 | 0.0752442 | 2.61E-05 |  | 2,836 | 374,441 | 0.0438315 | 0.0727616 | 0.546909 |
| 25 | rs142692233 | A | G | 0.331106 | 0.0810456 | 4.40E-05 |  | 2,836 | 374,441 | 0.133036 | 0.0785342 | 0.0902672 |
| 26 | rs144580248 | C | T | 0.20079 | 0.0488899 | 4.01E-05 |  | 2,836 | 374,441 | 0.0498107 | 0.0463526 | 0.282552 |
| 27 | rs145086244 | A | G | 0.883138 | 0.207972 | 2.17E-05 |  | 2,836 | 374,441 | -0.0773691 | 0.234213 | 0.741145 |
| 28 | rs147189267 | T | C | 0.465528 | 0.113527 | 4.12E-05 |  | 2,836 | 374,441 | 0.145669 | 0.122777 | 0.235444 |
| 29 | rs147667696 | T | C | -0.591326 | 0.145723 | 4.95E-05 |  | 2,836 | 374,441 | 0.177916 | 0.113809 | 0.117984 |
| 30 | rs147761221 | A | G | -0.437439 | 0.100985 | 1.48E-05 |  | 2,836 | 374,441 | -0.125729 | 0.0818982 | 0.12474 |
| 31 | rs147901527 | A | G | -0.289142 | 0.0700035 | 3.62E-05 |  | 2,836 | 374,441 | -0.00562052 | 0.0588093 | 0.923861 |
| 32 | rs148649817 | A | G | -0.374444 | 0.0874778 | 1.87E-05 |  | 2,836 | 374,441 | -0.126876 | 0.0687215 | 0.0648575 |
| 33 | rs150725653 | C | A | -0.444805 | 0.103859 | 1.85E-05 |  | 2,836 | 374,441 | 0.0683551 | 0.0799431 | 0.392526 |
| 34 | rs1536900 | C | T | -0.134555 | 0.030208 | 8.42E-06 |  | 2,836 | 374,441 | 0.00136466 | 0.0272645 | 0.96008 |
| 35 | rs17213068 | G | C | -0.252314 | 0.0548639 | 4.25E-06 |  | 2,836 | 374,441 | 0.00444485 | 0.0452169 | 0.921693 |
| 36 | rs17618440 | G | T | -1.13642 | 0.2796 | 4.81E-05 |  | 2,836 | 374,441 | -0.00847249 | 0.173179 | 0.960981 |
| 37 | rs17636116 | T | C | -0.350007 | 0.0852231 | 4.01E-05 |  | 2,836 | 374,441 | 0.0230837 | 0.0706925 | 0.744019 |
| 38 | rs187042189 | T | C | 0.290682 | 0.0706974 | 3.93E-05 |  | 2,836 | 374,441 | -0.0814787 | 0.0716646 | 0.255562 |
| 39 | rs200299868 | G | A | -0.230529 | 0.0556654 | 3.45E-05 |  | 2,836 | 374,441 | 0.0557245 | 0.052225 | 0.285968 |
| 40 | rs2033975 | T | C | -0.60286 | 0.145161 | 3.28E-05 |  | 2,836 | 374,441 | -0.00924267 | 0.102162 | 0.927913 |
| 41 | rs2081249 | T | C | -0.191369 | 0.0471167 | 4.87E-05 |  | 2,836 | 374,441 | 0.0377297 | 0.0446789 | 0.39841 |
| 42 | rs2225230 | C | T | -0.16423 | 0.0387108 | 2.21E-05 |  | 2,836 | 374,441 | -0.0243683 | 0.036193 | 0.500765 |
| 43 | rs2510737 | C | G | 0.12882 | 0.0309694 | 3.19E-05 |  | 2,836 | 374,441 | -0.006055 | 0.0281129 | 0.829469 |
| 44 | rs2637766 | G | A | -0.147341 | 0.0357007 | 3.67E-05 |  | 2,836 | 374,441 | -0.00147273 | 0.0309306 | 0.962024 |
| 45 | rs28823579 | T | G | -0.177824 | 0.0399014 | 8.33E-06 |  | 2,836 | 374,441 | -0.106178 | 0.0349242 | 0.00236396 |
| 46 | rs2993355 | G | A | -0.129075 | 0.031509 | 4.20E-05 |  | 2,836 | 374,441 | 0.00361747 | 0.0281655 | 0.897804 |
| 47 | rs35079494 | C | T | -0.157812 | 0.0381443 | 3.52E-05 |  | 2,836 | 374,441 | -0.0252172 | 0.0333635 | 0.44975 |
| 48 | rs35467279 | A | C | 0.146247 | 0.0320289 | 4.97E-06 |  | 2,836 | 374,441 | -0.00322158 | 0.0282351 | 0.90916 |
| 49 | rs367001 | T | G | -0.125527 | 0.0301593 | 3.15E-05 |  | 2,836 | 374,441 | -0.0405909 | 0.0269022 | 0.131342 |
| 50 | rs3857844 | G | C | 0.154322 | 0.0379508 | 4.78E-05 |  | 2,836 | 374,441 | 0.0140245 | 0.0349533 | 0.688246 |
| 51 | rs3956254 | T | C | -0.169196 | 0.0400637 | 2.41E-05 |  | 2,836 | 374,441 | -0.0679662 | 0.0350029 | 0.0521699 |
| 52 | rs4076019 | G | A | -0.126283 | 0.0300606 | 2.66E-05 |  | 2,836 | 374,441 | -0.0297132 | 0.0270039 | 0.271188 |
| 53 | rs4548693 | C | A | -0.155318 | 0.0337578 | 4.21E-06 |  | 2,836 | 374,441 | -0.0210991 | 0.0310026 | 0.496151 |
| 54 | rs4620012 | G | A | -0.138896 | 0.0326539 | 2.10E-05 |  | 2,836 | 374,441 | -0.0284579 | 0.0288034 | 0.32315 |
| 55 | rs4849528 | A | C | 0.170402 | 0.0419542 | 4.87E-05 |  | 2,836 | 374,441 | 0.0289784 | 0.0399069 | 0.467747 |
| 56 | rs4851628 | T | G | 0.130457 | 0.0314334 | 3.32E-05 |  | 2,836 | 374,441 | 0.030293 | 0.0285519 | 0.288698 |
| 57 | rs531586818 | G | C | -1.07311 | 0.253964 | 2.38E-05 |  | 2,836 | 374,441 | 0.212606 | 0.167379 | 0.20401 |
| 58 | rs56066057 | G | T | 0.127332 | 0.0310477 | 4.11E-05 |  | 2,836 | 374,441 | -0.0354406 | 0.0281328 | 0.207756 |
| 59 | rs57729369 | G | A | 0.267582 | 0.0634721 | 2.49E-05 |  | 2,836 | 374,441 | 0.0135885 | 0.0620585 | 0.826678 |
| 60 | rs6021953 | A | G | -0.168434 | 0.0346004 | 1.13E-06 |  | 2,836 | 374,441 | -0.0263085 | 0.0302154 | 0.383919 |
| 61 | rs6094353 | G | A | -0.268998 | 0.0603975 | 8.44E-06 |  | 2,836 | 374,441 | -0.0891105 | 0.0487896 | 0.067786 |
| 62 | rs61751012 | A | G | 0.60627 | 0.130456 | 3.36E-06 |  | 2,836 | 374,441 | 0.0824842 | 0.139591 | 0.554589 |
| 63 | rs62253086 | G | T | -0.311856 | 0.0763446 | 4.41E-05 |  | 2,836 | 374,441 | 0.0759514 | 0.0614213 | 0.216249 |
| 64 | rs6504128 | C | A | -0.330515 | 0.0806945 | 4.21E-05 |  | 2,836 | 374,441 | -0.0863992 | 0.0798065 | 0.278983 |
| 65 | rs6536404 | A | G | -0.131783 | 0.0307888 | 1.87E-05 |  | 2,836 | 374,441 | -0.0123895 | 0.0278486 | 0.656401 |
| 66 | rs6585536 | C | T | -0.131702 | 0.0309489 | 2.09E-05 |  | 2,836 | 374,441 | -0.0536399 | 0.0280685 | 0.0560003 |
| 67 | rs6855181 | C | T | -0.125118 | 0.030474 | 4.03E-05 |  | 2,836 | 374,441 | 0.0486165 | 0.0274343 | 0.0763783 |
| 68 | rs72788969 | T | C | 0.185977 | 0.0442647 | 2.65E-05 |  | 2,836 | 374,441 | -0.03887 | 0.0421301 | 0.356206 |
| 69 | rs72827609 | A | G | -0.650624 | 0.133413 | 1.08E-06 |  | 2,836 | 374,441 | -0.0929901 | 0.0940701 | 0.322898 |
| 70 | rs73142278 | T | C | 0.602307 | 0.131946 | 5.00E-06 |  | 2,836 | 374,441 | -0.0753333 | 0.142572 | 0.597231 |
| 71 | rs74549297 | G | A | 0.472855 | 0.113152 | 2.93E-05 |  | 2,836 | 374,441 | 0.0538509 | 0.12118 | 0.656762 |
| 72 | rs74863973 | C | T | -0.169312 | 0.0398096 | 2.11E-05 |  | 2,836 | 374,441 | -0.0343665 | 0.0345782 | 0.320282 |
| 73 | rs7502307 | G | C | 0.154206 | 0.0338021 | 5.07E-06 |  | 2,836 | 374,441 | -0.0243295 | 0.0309526 | 0.431853 |
| 74 | rs7503604 | A | C | -0.124948 | 0.0301801 | 3.47E-05 |  | 2,836 | 374,441 | 0.0105054 | 0.0270443 | 0.697683 |
| 75 | rs7530037 | C | T | -0.138833 | 0.0341907 | 4.90E-05 |  | 2,836 | 374,441 | 0.0107206 | 0.0310646 | 0.730014 |
| 76 | rs75758664 | A | C | 0.781302 | 0.187507 | 3.09E-05 |  | 2,836 | 374,441 | 0.327554 | 0.206783 | 0.113183 |
| 77 | rs76376367 | G | T | 0.652224 | 0.158696 | 3.96E-05 |  | 2,836 | 374,441 | 0.0642387 | 0.165794 | 0.698414 |
| 78 | rs76506460 | T | C | 0.48866 | 0.115024 | 2.15E-05 |  | 2,836 | 374,441 | 0.282535 | 0.110888 | 0.0108363 |
| 79 | rs769018 | T | C | 0.246554 | 0.0532497 | 3.65E-06 |  | 2,836 | 374,441 | -0.0254419 | 0.0446504 | 0.568812 |
| 80 | rs76993046 | C | T | -0.506649 | 0.122203 | 3.38E-05 |  | 2,836 | 374,441 | -0.0374488 | 0.0941406 | 0.69078 |
| 81 | rs7867845 | G | C | -0.199292 | 0.045819 | 1.36E-05 |  | 2,836 | 374,441 | 0.0307947 | 0.0399869 | 0.441228 |
| 82 | rs7918981 | A | G | 0.327741 | 0.0722265 | 5.69E-06 |  | 2,836 | 374,441 | 0.169891 | 0.0661922 | 0.010269 |
| 83 | rs79508047 | G | A | -0.254873 | 0.0568692 | 7.40E-06 |  | 2,836 | 374,441 | -0.0557893 | 0.0482094 | 0.24718 |
| 84 | rs79635473 | A | G | 0.272105 | 0.0612903 | 9.01E-06 |  | 2,836 | 374,441 | 0.0926673 | 0.058842 | 0.115291 |
| 85 | rs79817593 | T | C | -0.41989 | 0.102826 | 4.44E-05 |  | 2,836 | 374,441 | -0.145217 | 0.0804837 | 0.0711836 |
| 86 | rs80038448 | T | C | 0.213158 | 0.0457048 | 3.10E-06 |  | 2,836 | 374,441 | -0.0612338 | 0.0431694 | 0.156058 |
| 87 | rs8004419 | A | T | 0.137268 | 0.0318584 | 1.64E-05 |  | 2,836 | 374,441 | 0.0059208 | 0.0281483 | 0.8334 |
| 88 | rs868209 | A | T | -0.195814 | 0.0456582 | 1.80E-05 |  | 2,836 | 374,441 | -0.0526057 | 0.0385467 | 0.172339 |
| 89 | rs907238 | C | T | 0.326466 | 0.0676802 | 1.41E-06 |  | 2,836 | 374,441 | -0.124502 | 0.054659 | 0.0227389 |
| 90 | rs927170 | G | T | 0.21023 | 0.0505011 | 3.14E-05 |  | 2,836 | 374,441 | 0.0315003 | 0.047485 | 0.507092 |
| 91 | rs9463851 | G | A | -0.170269 | 0.0402399 | 2.32E-05 |  | 2,836 | 374,441 | 0.0165143 | 0.034634 | 0.633487 |
| 92 | rs955822 | T | C | -0.161672 | 0.0383737 | 2.52E-05 |  | 2,836 | 374,441 | -0.0221099 | 0.0335872 | 0.510356 |
| 93 | rs9971308 | G | A | 0.17341 | 0.0372816 | 3.30E-06 |  | 2,836 | 374,441 | 0.0616402 | 0.0326983 | 0.0594142 |

SNP, single nucleotide polymorphism; EA, effect allele; OA, other allele; SE, standard error; PTSD, post-traumatic stress disorder; GD, Graves' disease.

**Supplementary Table S3** The results of MR-Egger intercept analysis

| **Exposure** | **Outcome** | **Egger_intercept** | **SE** | ***p* value** |
| --- | --- | --- | --- | --- |
| PTSD | AIT | 0.016150074 | 0.022250372 | 0.469803273 |
| PTSD | GD | 0.009025197 | 0.0101772 | 0.377521314 |

*PTSD, post-traumatic stress disorder; AIT, Autoimmune thyroiditis; GD, Graves' disease.

**Supplementary Table S4** The results of heterogeneity analysis

| **Exposure** | **Outcome** | **Method** | **Q** | **Q_df** | **Q_*p* val** |
| --- | --- | --- | --- | --- | --- |
| PTSD | AIT | MR Egger | 94.19404855 | 91 | 0.388456646 |
| PTSD | AIT | Inverse variance weighted | 94.73937496 | 92 | 0.401668271 |
| PTSD | GD | MR Egger | 112.299997 | 91 | 0.064417519 |
| PTSD | GD | Inverse variance weighted | 113.2704961 | 92 | 0.065573901 |

*PTSD, post-traumatic stress disorder; AIT, Autoimmune thyroiditis; GD, Graves' disease.
